# Feasibility of vocal fold abduction and adduction assessment using cine-MRI

**DOI:** 10.1007/s00330-016-4341-3

**Published:** 2016-04-16

**Authors:** Marina Mat Baki, Alex Menys, David Atkinson, Paul Bassett, Simon Morley, Timothy Beale, Guri Sandhu, Georgekutty Naduvilethil, Nicola Stevenson, Martin A Birchall, Shonit Punwani

**Affiliations:** 1 Faculty of Medicine, National University of Malaysia, Kuala Lumpur, Malaysia; 2 University College London, Gower Street, London, WC1E 6BT UK; 3 Centre for Medical Imaging, University College London, 3rd Floor East, 250 Euston Road, London, NW1 2PG UK; 4 University College London Hospital NHS Trust, Royal National Throat Nose Ear Hospital, London, United Kingdom; 5 Imperial College Healthcare NHS Trust, Charing Cross Hospital, London, United Kingdom; 6 Department of Otolaryngology, University of California, Davis, Davis, CA United States; 7 Ear Institute, University College London, London, United Kingdom

**Keywords:** Vocal fold paralysis, Magnetic resonance imaging, Cine, Feasibility study

## Abstract

**Objective:**

Determine feasibility of vocal fold (VF) abduction and adduction assessment by cine magnetic resonance imaging (cine-MRI)

**Methods:**

Cine-MRI of the VF was performed on five healthy and nine unilateral VF paralysis (UVFP) participants using an axial gradient echo acquisition with temporal resolution of 0.7 s. VFs were continuously imaged with cine-MRI during a 10-s period of quiet respiration and phonation. Scanning was repeated twice within an individual session and then once again at a 1-week interval. Asymmetry of VF position during phonation (VF phonation asymmetry, VFPa) and respiration (VF respiration asymmetry, VFRa) was determined. Percentage reduction in total glottal area between respiration and phonation (VF abduction potential, VFAP) was derived to measure overall mobility. An un-paired t-test was used to compare differences between groups. Intra-session, inter-session and inter-reader repeatability of the quantitative metrics was evaluated using intraclass correlation coefficient (ICC).

**Results:**

VF position asymmetry (VFPa and VFRa) was greater (*p*=0.012; *p*=0.001) and overall mobility (VFAP) was lower (*p*=0.008) in UVFP patients compared with healthy participants. ICC of repeatability of all metrics was good, ranged from 0.82 to 0.95 except for the inter-session VFPa (0.44).

**Conclusion:**

Cine-MRI is feasible for assessing VF abduction and adduction. Derived quantitative metrics have good repeatability.

***Key points*:**

• *Cine-MRI is used to assess vocal folds (VFs) mobility: abduction and adduction.*

• *New quantitative metrics are derived from VF position and abduction potential.*

• *Cine-MRI able to depict the difference between normal and abnormal VF mobility.*

• *Cine-MRI derived quantitative metrics have good repeatability.*

## Introduction

The larynx has a pair vocal folds (VFs) that open (abduct) during respiration and close (adduct) during speech or phonation. Laryngoscopy either using 70 degree rigid telescope or flexible nasopharyngolaryngoscope is part of larynx examination in patients with voice disorders in the clinic. VF immobility caused by nerve injury or paralysis is detected during this routine scope examination.

Injury to the vagus nerve or its branch of the recurrent laryngeal nerve (RLN) causes vocal fold paralysis. Common aetiologies of vocal fold paralysis include iatrogenic (36 %), neoplastic (18 %), and idiopathic (18 %) in which unilateral is more common than bilateral paralysis (78 % and 22 %, respectively) [1]. Other less common causes are trauma, aortic aneurysm, radiation induced, and cardiovascular pathology. However, the commonest cause is iatrogenic including thyroid surgery, carotid endarterectomy, anterior approaches to the cervical spine, and heart or great vessel surgery [1]. Thyroidectomy has the highest incidence, and the trend may still be increasing despite improvement in surgical skill and use of intra-operative RLN monitor [1–4]. The injury could be temporary or permanent. Risk of permanent RLN palsy following thyroidectomy has been estimated at 1-2 % [5], but this may be a considerable underestimate due to problems of follow-up and reporting.

In VF paralysis, impaired abduction during respiration reduces the size of the glottal airway compromising breathing and affecting voice. However, presentations of vocal fold paralysis are different between unilateral and bilateral. The unilateral vocal fold paralysis (UVFP) patients are more concerned with voice rather than breathing as the contralateral normal vocal fold will still be abducting during respiration [6, 7], although they may have decreased effort tolerance, during strenuous activities. For the bilateral vocal fold paralysis (BVFP), the patients usually have problems with breathing, as both vocal folds are not abducting during respiration. Therefore, the aim of treatment for the UVFP is to achieve glottal closure during speech, whereas for the BVFP is to achieve increased in glottal opening during respiration.

In patients with BVFP, both VFs are commonly unable to abduct during inspiration causing obstruction to the airway. It may present with critical impairment of respiration [8–10]. Treatments for BVFP are crucial and may be life saving. They are mainly surgical aiming to increase the glottal airway to alleviate the respiratory compromise. Surgical options to enlarge the glottal airway are laser posterior cordotomy, arytenoidectomy, and laterofixation of the vocal fold with a suture and selective reinnervation [9–13]. Laser posterior cordotomy and arytenoidectomy either partial or total involves opening the posterior part of the glottis are static surgery that is not aiming for re-establishment of VF mobility. These surgical treatments have been reported as safe and effective in relieving airway obstruction, but with the expense of a hoarse, weak, and breathy voice. Selective reinnervation is a relatively new procedure to improve the glottal airway by re-establishing the VF abduction while preserving the VF tone and bulk for good voice quality [9, 12]. Outcome measures that have been used are Medical Research Council (MRC) breathlessness scale [14], which is subjective, airway resistance by performing lung function test, which is objective, but limited by the presence of tracheostomy or rate of decannulation for patients who had tracheostomy [15]. Therefore, objective measurement of the VF mobility is particularly useful in measuring effectiveness of selective reinnervation in re-establishing the VF abduction.

Quantification of VF mobility is necessary to provide a measure of effectiveness of interventions conducted to increase the glottal airway [16]. Whilst endoscopic direct visualisation is good nowadays, it remains technically difficult for objective measurements of the glottal airway. The correct measurements can only be achieved by maintaining the camera tangential to the plane of VFs with clear visualisation of the anterior commissure and vocal process [16]. This may be difficult to achieve in some patients. Glottal area assessed during respiration by flexible nasopharyngolaryngoscope can also be used to quantify VF abduction [16–18], but accuracy is limited by distortion caused by varying distance from the object and the wide angle lens of the endoscope [16–20]. To correct for this, the insertion of a probe to measure the distance from the tip of scope to the true VF has been suggested, but this increases the size of the endoscope required, is time-consuming, and uncomfortable for routine clinical application [21]. Imaging of the vocal folds with magnetic resonance imaging (MRI) may be used as an adjunct or a complement to laryngoscopy [22]. However, validated, non-invasive quantitative measures of vocal fold mobility assessed by MRI are not available.

In this study, we aimed to determine the feasibility of vocal fold (VF) mobility (abduction and adduction) assessment through cine magnetic resonance imaging (cine-MRI) derived quantitative metrics.

## Materials and Methods

The local ethics committee approved this study and informed consent was obtained from all participants.

### Participant selection

#### Healthy controls

From November 2012 until March 2013, five volunteers (two female ages 39 and 40 years, and three male ages 28, 31, and 35 years) without any current or previous history of vocal fold conditions (excluding previous upper respiratory tract infection) were recruited from the local hospital staff. As healthy controls had no current or past medical history of vocal problems, laryngoscopy to confirm the vocal fold mobility was not performed as an additional procedure within this cohort.

#### Unilateral vocal fold paralysis patients

From May 2012 until May 2013, nine UVFP patients (seven female, two male) with ages ranging from 19 to 62 years old were recruited from the local hospital ear nose and throat specialist voice clinics. Inclusion criteria were: (i) UVFP identified by flexible nasopharyngolaryngoscope (VNL-1590STi, KayPentax, USA) and confirmed by laryngeal electromyography; (ii) UVFP was present for more than 6 months of iatrogenic or idiopathic aetiology; and (iii) no surgical corrective procedures. Patients who were claustrophobic or had associated multiple lower cranial nerve palsies, VF lesions, voice related neurological disorders (spasmodic dysphonia, voice tremor), or cricoarytenoid joint fixation, or had any implants in the neck region that may cause substantial image artefacts were excluded from the study.

### Cine-MRI

#### Imaging Protocol

All participants were imaged on a 3T Philips Achieva TX MRI scanner (Philips Healthcare, the Netherlands).

Sense Flex S coils of 11 cm diameter were placed at the left and right sides of the neck covering the anterior part of the larynx below the angle of the mandible, while the participant lay supine on the scanner bed with the head resting on a headrest. The coils were held steady by placing a neck strap and sandbags at the side of the head and neck to minimize movement.

**Fig. 1:**
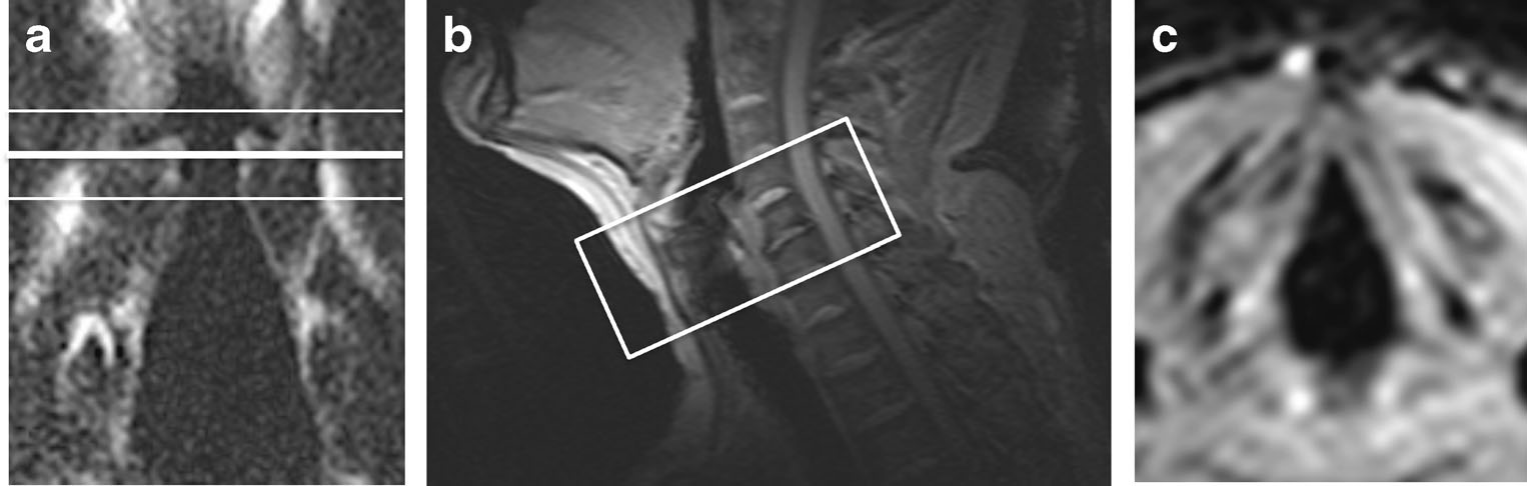
**a**) A T2-weighted coronal view of a participant showing three slices placement, two *white lines* at the top and bottom and one *bold line* in the middle through the superior surface of the true vocal folds. **b**) A sagittal reference scan showing the angulation of the axial motion sequence positioned at right angles to the larynx. **c**) A frame from an axial motion sequence image of vocal folds during respiration

Details of the protocol used for the MRI are summarized in Table 1. Sagittal reference images were used to angle the axial cine-MRI slices parallel to the vocal folds. Subsequently, a T2-weighted turbo spin echo coronal acquisition was performed to cover the larynx. Three cine-MRI axial slices were then positioned to ensure that the vocal folds remained within the imaging volume during the normal vertical elevation that occurs on phonation (Fig. 1).

**Table 1:** MRI protocol for vocal fold motion and coronal T2-weighted imaging

	Vocal fold motion	T2-weighted
Sequence Name	Fast field echo (FFE)	SE-TSE
Repetition time (ms)	2.1	6789
Echo time (ms)	0.82	120
Image resolution	1.5x1.5	0.5x0.5
Slice thickness (mm)	10	2.5
Number of slices	3	14
Flip angle	20	90
Field of view (FOV) (mm)	240x180	140x140
Temporal resolution (s)	0.764	-
Total acquisition time	1 min 31 s	5 min 32 s
Number of dynamic scans	120	-

#### Phonation and Respiration Tasks

Prior to imaging, participants were instructed in, and practiced, a standardised set of phonation tasks [22]. Specifically, they were asked to phonate /hee/ for the first 10 s of scanning, then breathe quietly through the nose for 10 s; phonate /hee/ for a further 10; breathe quietly again for 10 s; and then phonate /hee/ for a final 10 s. Ten sequential cine-MRI acquisitions were performed during each /hee/ and quiet breathing period. Participants were instructed to perform these standardized tasks during cine-MRI acquisition.

The complete dataset for each participant comprised two intra-session cine-MRI studies (study 1 and study 2), and for all healthy volunteers and five of the nine UVFP patients a third inter-session cine-MRI study (study 3) performed at a 1-week interval. Each study comprised 10 sequential cine-MRI acquisitions performed during phonation and a second set of 10 sequential cine-MRI acquisitions performed during respiration.

### Cine-MRI parameters of vocal fold mobility

Here we described two methods of deriving Cine-MRI parameters using 1) glottal area (introduced by the present research) and 2) glottal angle (introduced by Ahmad et al. [23]).

### Parameters using glottal area

**Fig. 2:**
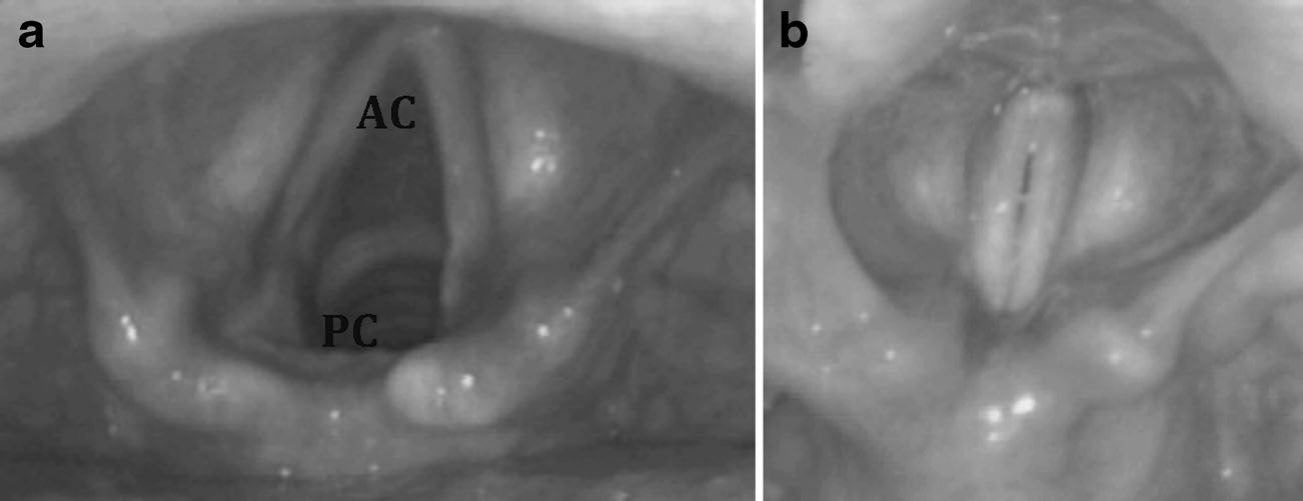
This endoscopic view of the larynx depicts the vocal folds position during **a**) respiration and **b**) phonation. *AC,* Anterior commissure; *PC*, Posterior commissure

**Fig. 3:**
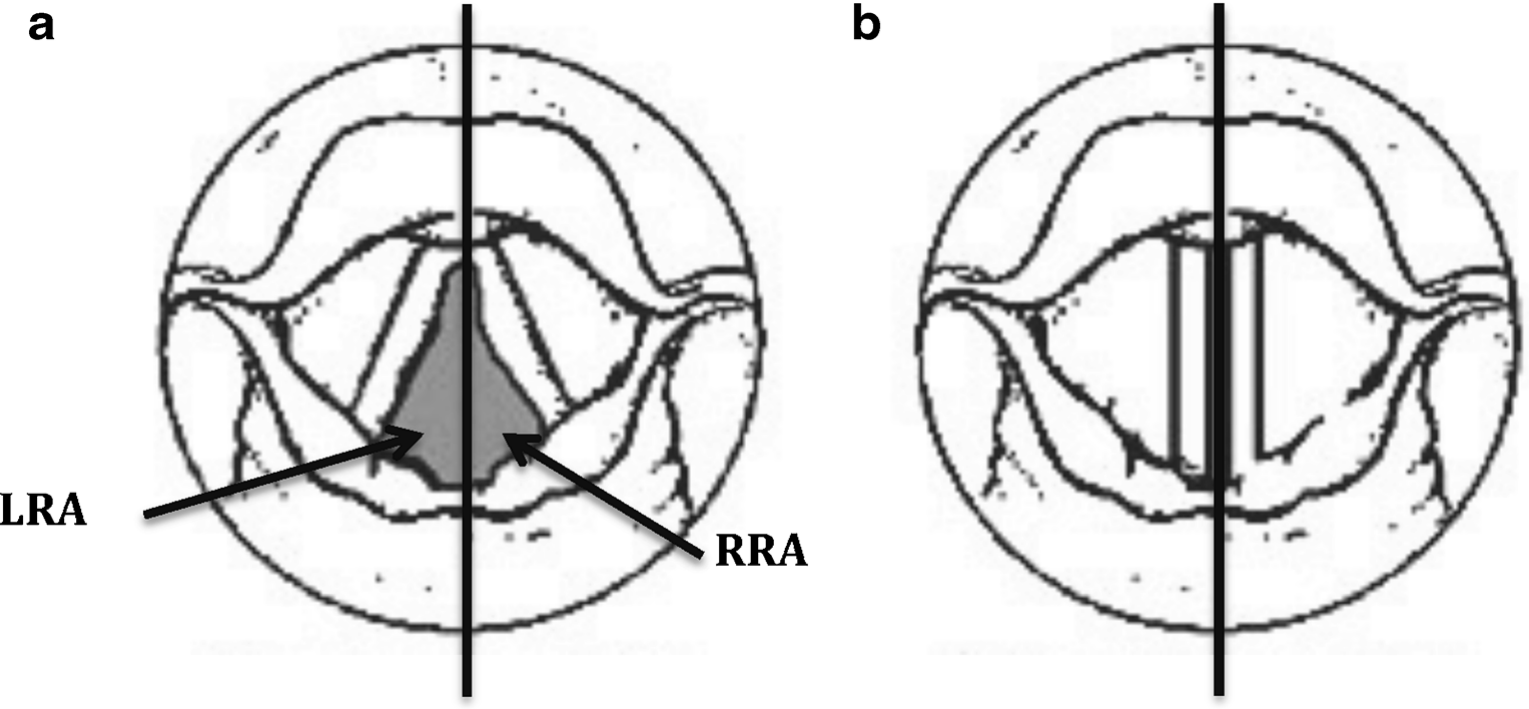
Schematic diagram depicting an imaginary line bisecting the anterior and posterior commissure and dividing the **a**) respiration and **b**) phonation area into right and left. Note that the phonation area is very small. *LRA*, Left sided hemi-glottal area during respiration; *RRA*, Right sided hemi-glottal area during respiration

**Fig. 4:**
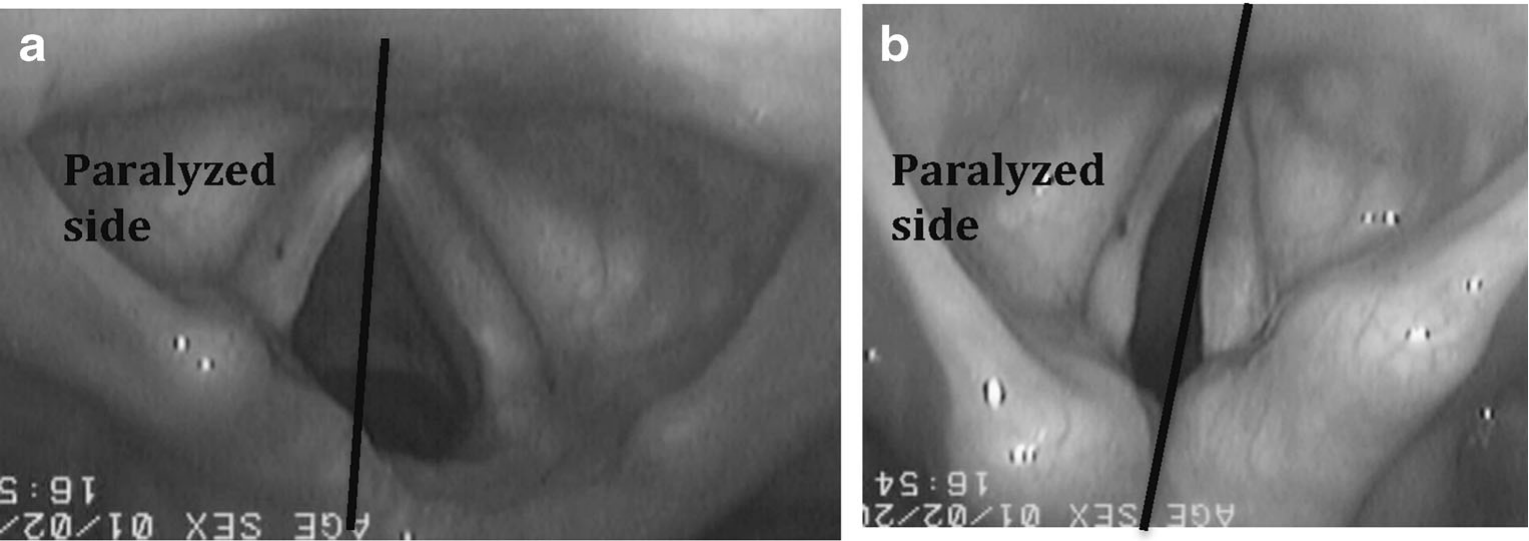
This endoscopic view of left vocal fold paralysis depicts the position of the paralysed vocal folds during **a**) respiration and **b**) phonation

Normal VFs symmetrically abduct and adduct during respiration and phonation as shown in Fig. 2. Image-based metrics were defined to reflect this mobility, and calculated through changes in glottal area. Glottal area is the open airway area visualized on axial views of the vocal folds, bounded on the right and left by the medial borders of the VFs, anteriorly by the anterior commissure and posteriorly by the posterior commissure. A line originating from the anterior commissure and bisecting the glottal area creates left and right hemi-glottal areas (Fig. 3). Incomplete abduction of a vocal fold results in asymmetry between left and right hemi-glottal areas. Therefore, in principle a ratio of left and right hemi-glottal area (R) deviant from unity reflects asymmetric vocal fold movement (R>1 reflecting left sided paralysis; R<1 reflecting right sided paralysis), and magnitude of the deviance from 1 reflects the severity of effect. This phenomena is recognized during direct laryngoscopic visualization of the VFs (Fig. 4) [24].

Three quantitative metrics derivable from cine-MRI and reflecting vocal fold mobility are defined. VF phonation asymmetry (VFPa) and VF respiration asymmetry (VFRa) is defined as the 1-R during phonation and respiration respectively, reflecting the magnitude of asymmetric VF movement. VF abduction potential (VFAP) is defined as the percentage change in total glottal area occurring from respiration to phonation and provides a global assessment of VF mobility.

### Parameters using glottal angle

Glottal angle is an angle between the right and left vocal folds and the anterior commissure as described by Ahmad et al. All the calculation of parameters mentioned above was repeated using the left and right glottal angle instead of glottal area. And these parameters were marked as ‘*’, for example *VFPa.

### Image analysis

Images were downloaded to an OsiriX (open-source software, Geneva, Switzerland) [25] workstation for analysis.

For each participant, two readers (MB.M. and A.M.), independently performed measurements on the cine-MRI acquired for study 1. A single reader (MB.M.) performed repeat measurements of the same patients on study 2 and study 3.

### Image analysis using glottal area

**Fig. 5:**
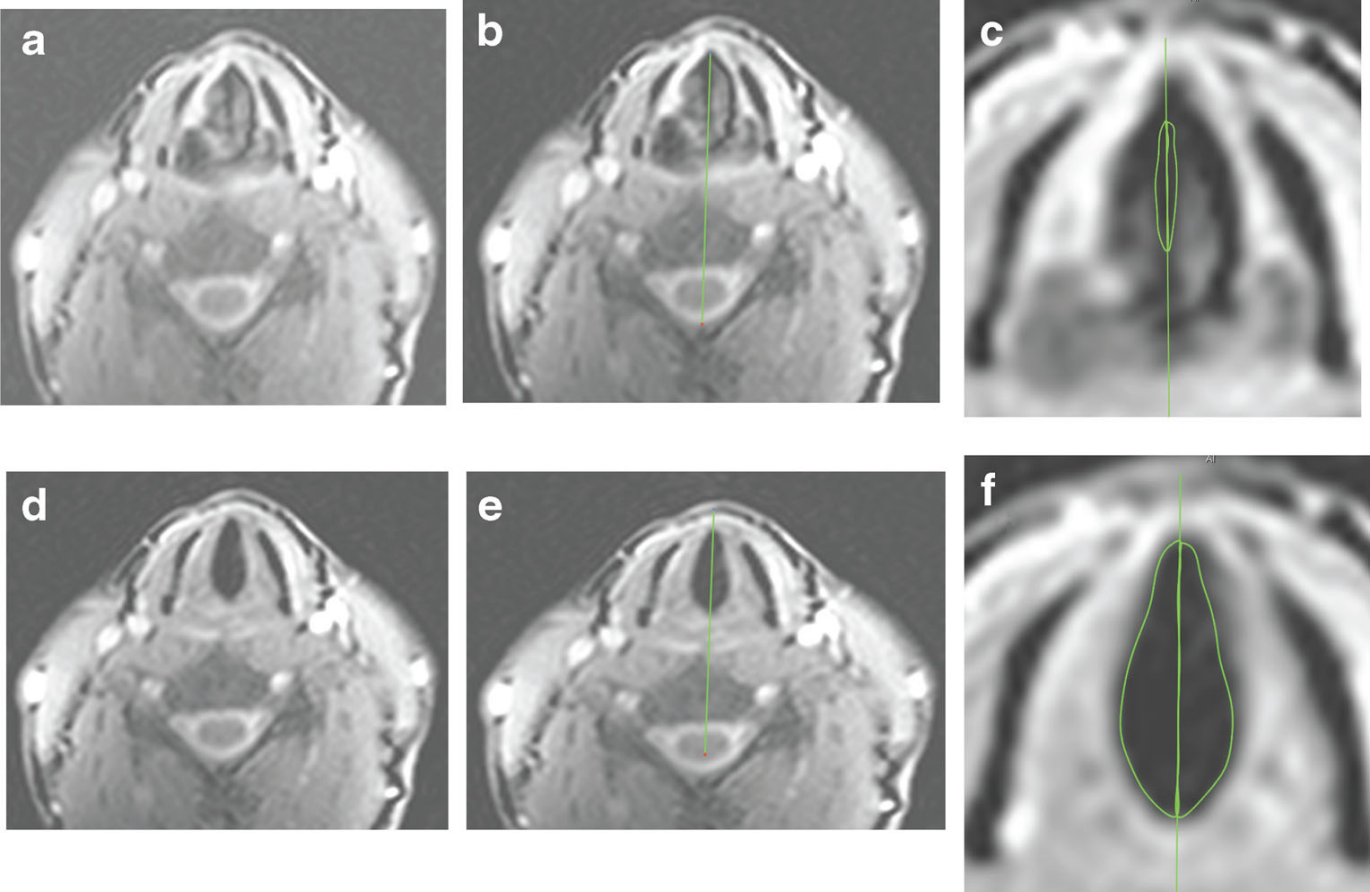
**a**, **d**: selected images showing glottal area during phonation (**a**) and respiration (**d**); **b**, **e**: A *vertical line* was drawn from anterior commissure bisecting the posterior commissure and the cervical vertebra dividing the triangular larynx into left and right glottal areas; **c**, **f**: Regions of interest (ROI) drawn to measure left and right phonation (**c**) and respiration (**f**) areas

**Fig. 6:**
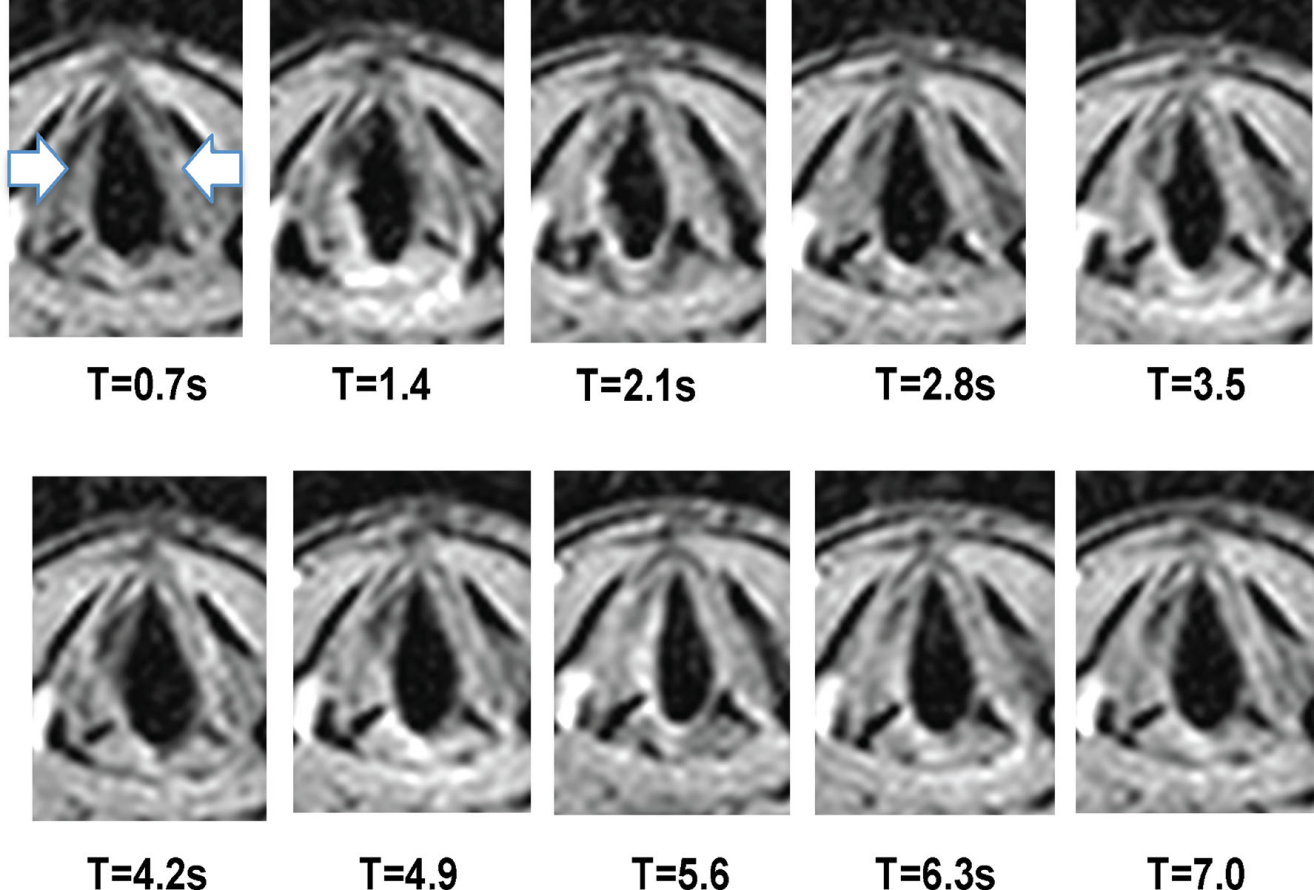
Selected sequential images of respiration at 10 time points of a healthy volunteer. The *arrows* show right and left vocal folds

The axial cine-MRI slice that best depicted the glottal area boundaries was selected (Figs. 5a, d, and 6). A vertical line was drawn from the anterior commissure bisecting the posterior commissure and the cervical vertebra dividing the triangular larynx into left and right halves (Fig. 5b and e). A region of interest was then used to measure the left and right hemi-glottal areas between the bisecting medial edge of the respective VF (Fig. 5c and f). Area measurements were repeated on each sequential image acquired during a 10-s period of phonation or respiration. Mean areas across sequential images were calculated for the phonation and respiration periods. Total glottal area was calculated by the sum of the left and right hemi-glottal areas. The extracted area measurements were used to calculate VFPa, VFRa, and VFAP as defined earlier.

### Image analysis using glottal angle

As mentioned above, a similar vertical line was drawn. The left and right *hemi-glottal angle was measured between the vertical line and the left and right vocal folds, respectively. All analyses were done similarly using the glottal angle instead of glottal area.

### Statistical analysis

Study derived metrics (VFPa, VFRa, and VFAP) were compared between healthy volunteers and UVFP using an un-paired t-test. Significance was defined at *p*<0.05. Intra-session (study 1 verses 2 within the same scan session), inter-session (study 1 verses 3 within separate scan sessions), and inter-reader repeatability (study 1) of study derived metrics was assessed using the intraclass correlation coefficient test (ICC) [26]. ICC was considered almost perfect for ICC values of 0.81 to 1.00, substantial for ICC values of 0.61 to 0.8 and moderate for ICC values of 0.4 to 0.6 [27].

All of this statistical analysis was repeated for study derived metrics using glottal angle (*VFPa, *VFRa, and *VFAP).

## Results

### Cine-MRI parameters using glottal area

**Fig. 7:**
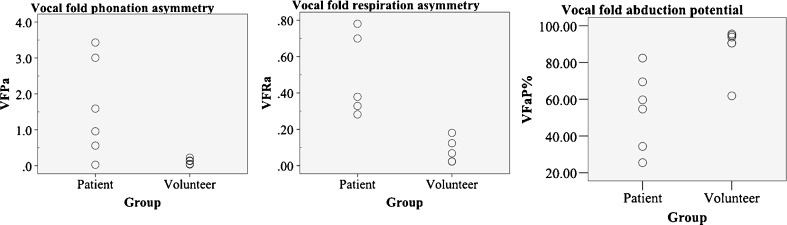
Graphs showing distribution of VFPa, VFRa, and VFAP in left vocal fold paralysis patients and healthy volunteers

VFPa and VFRa were significantly higher for UVFP patients compared with healthy volunteers (*p*<0.05), indicating asymmetrical mobility of the VF in UVFP patients during phonation and respiration (Fig. 7). Mean (±SD) VFPa of healthy volunteers was 0.08 ± 0.05, 0.12 ± 0.08 and 0.17 ± 0.13 for studies 1, 2, and 3, respectively. Mean (±SD) VFPa of UVFP patients were 1.13 ± 0.85, 1.28 ± 1.2, and 0.43 ± 0.41 for studies 1, 2, and 3, respectively. Mean (±SD) VFRa of healthy volunteers was 0.08 ± 0.07, 0.06 ± 0.07, and 0.10 ± 0.10 for studies 1, 2, and 3, respectively. Mean (±SD) VFRa of UVFP patients were 0.47 ± 0.20, 0.50 ± 0.21, and 0.73 ± 0.08 for studies 1, 2, and 3, respectively.

VFAP was significantly reduced in patients with UVFP compared with healthy volunteers (*p*<0.05), reflecting the reduced change in glottal area between respiration and phonation tasks due to VF paralysis (Table 2).

**Table 2:** Mean and standard deviation of VFPa, VFRa, and VFaP of healthy volunteers and patients measured for study 1, study 2, and study 3

Metrics	Readers	Study 1 (first 10 sequential images of within the same scan)		*p* values of comparison between volunteers and patients	Study 2 (second 10 sequential images of within the same scan)		Study 3 (first 10 sequential images of the second visit scan)	
		Volunteer (n = 5)	Patient (n =9)		Volunteer (n = 5)	Patient (n =9)	Volunteer (n = 5)	Patient (n =5)
VFPa	Reader1	0.08 (0.05)	1.13 (0.85)	0.012	0.12 (0.08)	1.28 (1.20)	0.17 (0.13)	0.43 (0.41)
	Reader2	0.22 (0.16)	0.99 (0.73)		N/A	N/A	N/A	N/A
VFRa	Reader1	0.08 (0.07)	0.47 (0.20)	0.001	0.06 (0.07)	0.50 (0.21)	0.10 (0.10)	0.73 (0.08)
	Reader2	0.16 (0.09)	0.58 (0.18)		N/A	N/A	N/A	N/A
VFAP	Reader1	87.32 (14.35)	57.61 (18.10)	0.008	82.48 (19.26)	52.85 (19.68)	92.14 (5.22)	72.79 (12.76)
	Reader2	83.35 (17.85)	51.64 (22.22)		N/A	N/A	N/A	N/A

VFPa: Vocal fold phonation asymmetry

VFRa: Vocal fold respiration asymmetry

VFAP: Vocal fold abduction potential

N/A = not applicable

Table 3 summarizes the repeatability statistics of derived metrics. There was an almost perfect intra-session ICC for VFPa, VFRa, and VFAP of 0.92, 0.95, and 0.90, respectively. Similarly, inter-session ICC of VFRa and VFAP were 0.94 (0.78, 0.98) and 0.82 (0.62, 0.97), demonstrating almost perfect agreement between temporally separated scan sessions. However, the inter-session ICC of VFPa was of 0.44 (-0.24, 0.83), which is moderate. Inter-reader ICC was almost perfect: 0.84, 0.82, and 0.91 for VFPa, VFRa, and VFAP, respectively, supportive of almost perfect agreement of metrics calculated from the same dataset by two independent readers.

**Table 3:** Results of intra-session, inter-scan, and inter-reader repeatability for cine-MRI parameters using glottal area and glottal angle

Metrics	Intra-session	Inter-scan	Inter-reader
VFPa	0.92 (0.76, 0.97)	0.44 (-0.24, 0.83)	0.84 (0.66, 0.93)
VFRa	0.95 (0.85, 0.98)	0.94 (0.78, 0.98)	0.82 (0.60, 0.92)
VFAP	0.90 (0.69, 0.97)	0.82 (0.62, 0.97)	0.91 (0.77, 0.96)
*VFPa	0.98 (0.94, 0.99)	0.62 (0.03, 0.89)	0.46 (0.14, 0.70)
*VFRa	0.78 (0.45, 0.92)	0.85 (0.52, 0.96)	0.65 (0.34, 0.83)
*VFAP	0.97 (0.91, 0.99)	0.78 (0.35, 0.94)	0.62 (0.35, 0.94)

Results presented are ICC values and the 95 % confidence interval in the bracket. The ‘*’ represents parameters derived from glottal angle

### Cine-MRI parameters using glottal angle

*VFRa was significantly higher (*p*<0.05) for UVFP patients (mean ± SD: 0.34 ± 0.23) compared with healthy volunteers (0.05 ± 0.06). However, although *VFPa of UVFP patients (0.87 ± 1.21) was higher than healthy volunteers (0.08 ± 0.12), it was not statistically significant (*p*=0.18). For VFAP, it was significantly reduced (*p*<0.01), in patients with UVFP (42.11 ± 37.78) compared with healthy volunteers (*p*<0.05.

Table 3 summarizes the repeatability statistics of derived metrics of glottal angle. There was a substantial to almost perfect intra-session ICC for *VFPa, *VFRa, and *VFAP of 0.98, 0.78, and 0.97, respectively. Similarly, inter-session ICC of *VFPa, *VFRa and *VFAP were 0.62, 0.85, and 0.78, demonstrating substantial to almost perfect agreement between temporally separated scan sessions. Inter-reader ICC was substantial: 0.65 and 0.62 for *VFRa and *VFAP, respectively, but moderate at 0.46 for *VFPa.

## Discussion

Objective measurement of glottal airway is important in clinical assessment of vocal fold paralysis and necessary for monitoring new therapeutic interventions to improve VF mobility (abduction and adduction). The cine-MRI in this study was to minimise motion artefact and was not meant to capture vocal fold vibrations during phonation that is ranged 70 to 500 Hz in males and 130-1000 Hz in females [28], which is too fast to be acquired by the cine-MRI at the moment, but can be captured by the video laryngostroboscopy. However, the main aim in our study is to capture VF mobility in abduction and adduction. The vocal folds adduct to the mid-glottal during phonation to allow vibration of the vocal folds by air from the lungs to produce voice whereas for breathing, the vocal folds abduct away from the mid-glottal to allow air transmission.

Within this study we assessed the feasibility of cine-MRI to provide non-invasive objective quantitative metrics of vocal fold mobility in abduction and adduction. We evaluated three metrics based on changes in glottal area (VFPa, VFRa, VFAP) and confirmed their feasibility and repeatability through application in UVFP and healthy volunteer cohorts. Clinical assessments of mobility in terms of abduction and adduction were done by performing laryngoscopy in patients to confirm paralysis in which all had one-sided paralysis. Therefore, the opposite normal side acts as internal controls as well. In addition, we also evaluated similar metrics, but based on changes in glottal angle (*VFPa, *VFRa, *VFAP). Results of glottal angle presented here are mainly to show the reliability of these parameters in comparison to the glottal area based parameters.

In theory, normal VF mobility should result in symmetric abduction during respiration and symmetric adduction during phonation. We, therefore, hypothesized that asymmetry in hemi-glottal airway area at the level of the VFs measured on cine-MRI could provide a quantitative marker of VF paralysis, and that changes in total glottal area between respiration and phonation could provide a global measure of abduction potential. We found that it was feasible to assess VF mobility with cine-MRI, that quantitatively derived metrics were significantly different between UVFP patients and healthy volunteers, and that these metrics had almost perfect repeatability.

Previous work in cine-MRI of the larynx to study vocal fold mobility involved a small series. A small number of paediatric patients with mixed airway disorders showed that cine-MRI of the larynx correlates with endoscopic findings [29]. In the study, UVFP was identified correctly on the cine-MRI. However, only one UVFP patient included in the study and the mobility of VF was evaluated subjectively. In another study, radiologists have used 1.5T cine-MRI as a clinical tool to evaluate 12 consecutive patients presenting with hoarseness and were able to correctly qualitatively diagnose impaired VF mobility on images acquired on coronal planes [22]. However, there were no healthy controls included in the study. There are limited publications on quantitative evaluation of VF mobility. To the best of our knowledge, only one previous study has quantitatively assessed VF mobility, assessing horizontal displacement of the VFs on axial planes during phonation, which may represent VF vibration in healthy volunteers [23]. In this study, Ahmad et al. explored the physiology of speech by measuring the glottal angle at the anterior commissure at different time point during phonation using three different vowels (/hee/, /haa/, and /huu/). Our study expands this work through defining new quantitative metrics and assessing their repeatability and feasibility for determining differences in VF mobility (abduction and adduction) between healthy volunteers and UVFP patients. We also evaluated the repeatability of metrics derived from the glottal angle as described by Ahmad et al. in which, between glottal area and glottal angle based metrics, the present study showed better repeatability results for the glottal area for the mentioned purposes.

Our fast cine-MRI was designed to capture of abduction/adduction of the vocal folds between respiration and phonation. The temporal resolution of our cine-MRI protocol was 0.7 s and is comparable to that published by Schlamann et al. [22]. Other authors have not explicitly defined this parameter within the manuscript [23, 29].

We acquired cine-MRI both during respiration and phonation in order to assess the change from VF abduction to adduction. We expected VFAP to be 100 % for healthy volunteers and 50 % in patients with UVFP. Indeed, we confirmed a significantly higher (*p*<0.05) mean VFAP of 87 % for healthy volunteers compared with 57 % for UVFP patients. Also, as expected, we confirmed significantly lower asymmetry indices (VFPa and VFRa) in the healthy volunteer cohort (mean values 0.08 to 0.22) compared with the UVFP patients (mean values 0.99 to 1.13).

Repeatability of quantitative metrics is a key determinant of clinical feasibility. Except for the inter-session repeatability of VFPa (ICC=0.44); intra-session, inter-session, and inter-reader repeatability of all three study metrics (VFPa, VFRa, and VFAP) had almost perfect repeatability (ICC>0.8). The moderate inter-session repeatability of VFPa is likely to be related to positioning of the axial imaging slices. We believe that this is the cause of the variation in measurement between patients and repeated studies, which affects especially the measurement during phonation (VFPa). Furthermore, previous studies have shown that during phonation, the normal larynx elevates [23], and paralysed VFs lack of the vertical elevation [29], which may compromise area assessment on a given axial image and cause variation between repeated studies. Despite this effect, other quantitative metrics evaluated within this study appear relatively robust to it. Repeatability may potentially be effected by the vowel sound used. Therefore, employing different vowel sounds such as (/haa/ or /huu/) may potentially be more repeatable as previous MRI data had demonstrated lower larynx position during these phonation tasks [23, 30].

Although limited by a small number of participants, the present study depicts the feasibility of quantifying VF mobility using cine-MRI, and indicates capability of cine-MRI measures to identify UVFP. Larger number of patients is necessary for future studies to confirm the present study’s encouraging results. A further limitation is that the study does not address generalisability of the technique across MRI scanners or across centres; however, we expect reproducibility across centres to be good, as the technique relies on simple, albeit rapid, anatomical MRI readily available on standard clinical scanners.

In summary, our study demonstrates that cine-MRI can objectively quantify VF mobility (abduction and adduction) and detect UVFP by derived quantitative metrics. With further validation this technique has the potential to provide an objective non-invasive measure of treatment outcome in clinical trials of surgical interventions that specifically aim for re-establishment of vocal fold mobility [12, 31].
